# Fuzzy-clustering and fuzzy network based interpretable fuzzy model for prediction

**DOI:** 10.1038/s41598-022-20015-y

**Published:** 2022-09-29

**Authors:** Xiaowei Wang, Yanqiao Chen, Jiashan Jin, Baohua Zhang

**Affiliations:** 1 College of Power Engineering, Naval University of Engineering, Wuhan, 430033 China; 2 Army Engineering University of PLA, Wuhan, 430075 China

**Keywords:** Applied mathematics, Computational science, Information technology

## Abstract

Interpretability is the dominant feature of a fuzzy model in security-oriented fields. Traditionally fuzzy models based on expert knowledge have obtained well interpretation innately but imprecisely. Numerical data based fuzzy models perform well in precision but not necessarily in interpretation. To utilize the expert knowledge and numerical data in a fuzzy model synchronously, this paper proposed a hybrid fuzzy c-means (FCM) clustering algorithm and Fuzzy Network (FN) method-based model for prediction. The Mamdani rule-based structure of the proposed model is identified based on FCM algorithm from data and by expert-system method from expert knowledge, both of which are combined by FN method. Particle swarm optimization (PSO) algorithm is utilized to optimize the fuzzy set parameters. We tested the proposed model on 6 real datasets comparing the results with the ones obtained by using FCM algorithm. The results showed that our model performed best in interpretability, transparency, and accuracy.

## Introduction

Fuzzy model is to implement logical reasoning and intelligent calculation for fuzzy information without a definite mathematical model. The main method is a set of IF–THEN rules-based procession designed either from expert knowledge or numerical data. Although independent on accurate mathematical model, fuzzy model is a powerful technique for logical reasoning, numerical calculation and non-linear function approximation^1^.

To identify a fuzzy model, structure establishment and parameter estimations are two main steps^2^. Structure identification is related to the number of rules after the important input variables have been selected. Parameter estimation (fuzzy set parameters) descripts a reliable non-linear approximation system.

Interpretability is the major feature of a fuzzy model in special fields, for example in security-oriented fields, and military field. There is a large amount of knowledge on top of the experience of experts. When this kind of knowledge is dealt with by machine learning, the security of this fuzzy logic (FL) model is vital for military application, and also fundamental for the transparency and interpretability of the knowledge. This kind of knowledge expressed by FL is conceptually easy to understand, tolerant of the imprecise information, and easy for human communication. Thus, expert knowledge based fuzzy models are traditionally well interpretable^3^, which is the motivation of the approach with fuzzy logic.

Expert knowledge is mainly expressed in Mamdani-type IF–THEN rules because this structure is more interpretable than T-S-type ones. Arguably, traditionally fuzzy models based on expert knowledge have obtained well interpretable innately. So, Mamdani rule-based structure is suitable for an interpretable-oriented fuzzy model.

Numerical data based fuzzy models are not necessarily interpretable, but lots of them perform well in precision with the help of soft computing techniques. For example, artificial Neural network (ANN), inspired by neuroscience, is one of the most successful methods in “learning” from data. Modern ANNs, particularly “deep learning” models, have been sped up by the increase of raw computer power. But “they cannot approach the cognitive capabilities of a four-year old. Perhaps more striking is that ANNs remain even further from approaching the abilities of simple animals^4^.” It implies that the innate structure plays the dominant role in the learning capability.

The FCM clustering algorithm is a powerful unsupervised learning technique to form a few rules with simple and interpretable structure^5^. FCM induces rules by organizing and categorizing data into partitions. Partitions with homogeneous data form clusters, and each cluster is associated with a rule. The fuzzy sets of rules are independent from each other. Each dimension of data is tailored only for one rule^6^. In order to improve the precision, many optimization-based FCM models have been proposed combining with metaheuristic optimization algorithms, such as genetic algorithm(GA)^7,8^ and PSO^9–15^. Both GA and PSO aim to solve optimization problems without being trapped into local minima. Due to its versatility and simplicity, PSO has become one of the most popular metaheuristics and an important tool for many applications.

The fuzzy models for prediction should utilize the expert knowledge and numerical data in a fuzzy model synchronously^16,17^. Historical data provides numerical quantitative measurements from past projects regarding the internal and external quality attributes. Experts utilize their experience to provide “fuzzy” information, or qualitative descriptions of the correlation between the internal and external quality attributes^18^. To make the model for prediction more practical, interpretability is crucial as it allows the practitioners to provide their own judgment on the predictors in linguistic terms. Black box prediction models (e.g., ANN-based models), are hard to identify the structure and to incorporate the experts’ judgments.

The novel method of FN is capable to combine the rules both from knowledge and from data. As a kind of Chained Fuzzy System (CFS)^19^ or Hierarchical Fuzzy System (HFS)^20,21^, FN maps the inputs to the outputs by means of connections^22–24^. The overall number of rules in FN is a linear function of the number of inputs and the number of linguistic terms per input. Compared to Standard Fuzzy System (SFS), the rules in FN are reduced and simplified. The structure in FN is more transparent and interpretable. Arguably, FN is characterized by interpretable innately as a white-box model. The details about FN will be introduced in section “Related methods”.

This paper proposes a hybrid FCM and FN based model for prediction (FCM-FN). FCM-FN is Interpretability-oriented model with Mamdani rule-based structure. The rules are generated by FCM method from data in the first step, and then by expert-system method from expert knowledge in the second step. Afterwards, the rules in both previous steps are connected by FN method. The Interpretable structure of FCM-FN model is identified. As a kind of prediction model, FCM-FN pursues accuracy while preserving interpretable structure. PSO algorithm is utilized to optimize the fuzzy set parameters which is initialized by FCM algorithm in the first step, and by expert system in the second step. For simplicity, the FCM-FN is a kind of multi-input single-output (MISO) type-1 fuzzy system. A multi-input multi-output (MIMO) fuzzy system can be taken as the composition of several MISO fuzzy systems^25^. As for type-2 fuzzy system, the related theories and practices have achieved diverse developments^26^, but the interpretability is rarely included into the type-2 theory so far. This is the reason why the FCM-FN is limited in type-1 structure in this paper.

The rest of the paper is synthesized as follows: section “Related methods” describes the Mamdani’s fuzzy model, FCM method, PSO method and FN theory, which will be used in this paper. In section “The proposed fuzzy model for prediction” the structure of the proposed FCM-FN model is analyzed in detail. The effectiveness of the model is illustrated in section “Case study”, through applications to various real datasets. The paper ends with the concluding remarks in section “Conclusion”, where the proposed approach is summarized, and its main characteristics are identified.

## Related methods

In this section, the related methods mentioned in the previous section are now further explained. They are divided in five parts: Mamdani-type fuzzy inference, FCM, PSO, PSO based tuning membership functions, and FN.

### Mamdani-type fuzzy inference

Inspired by Zadeh^27,28^, one of the most interpretable fuzzy models was suggested^29^, in which Mamdani attempted to control a steam engine and boiler combination by synthesizing a set of linguistic rules from experienced human operators (expert knowledge). The literature on the Mamdani-type fuzzy logic has grown rapidly. One of them was implemented as an universal approximator^17^. Mamdani-type fuzzy inference includes four steps. In order to explain Mamdani-type fuzzy inference, the example^17^ is employed where the *M* rules are formed as Eq. (1).

1 $$R_{j} : \, if\;x_{1} \;is\;A_{1}^{j} \;and \ldots and\;x_{n} \;is\;A_{n}^{j} \;then\;z\;is\;B^{j} ,$$

where j = *1,2,…,M, x*_*i*_*(i* = *1,2,…,n)* are the input variables to the fuzzy system, *z* is the output variable, $A_{i}^{j}$ and *B*^*j*^ are linguistic terms of the linguistic variables *x*_*i*_ and *z* in the universes of discourse *U* and *R*, respectively.

#### Fuzzification

The first step is to take the crisp inputs, and determine the degree to which these inputs belong to each of the appropriate fuzzy sets. Membership functions associating weighted inputs define functional overlaps between inputs, and ultimately determine output responses. Because membership functions are graphical representations of the magnitude of input participations in fuzzy logic, a fuzzy set is defined by its membership functions. Let *X* be a set of items, known as the universe, and its elements are denoted by *x*. And, a fuzzy subset A in *X* is characterized by the membership function *μ*_*A*_*(x)* which is associated with each element *x* in A and a real number in the interval [0, 1]. The membership function *μ*_*A*_*(x)* maps each element *x* to a membership value, which represents the level of membership of *x* in A. Different membership functions can be associated with different inputs and outputs. In essence, they are weighting factors for the outcomes of fuzzy rules. Gaussian or triangular shape are two well-known membership functions.

Gaussian membership function is specified by two parameters as Eq. (2).

2 $$\mu_{A} (x) = \exp \left[ { - \frac{1}{2}\left( {\frac{x - \theta }{\sigma }} \right)^{2} } \right],$$

where* θ* is the position of the peak relative to the universe, *σ* is the standard deviation.

**Figure 1:**
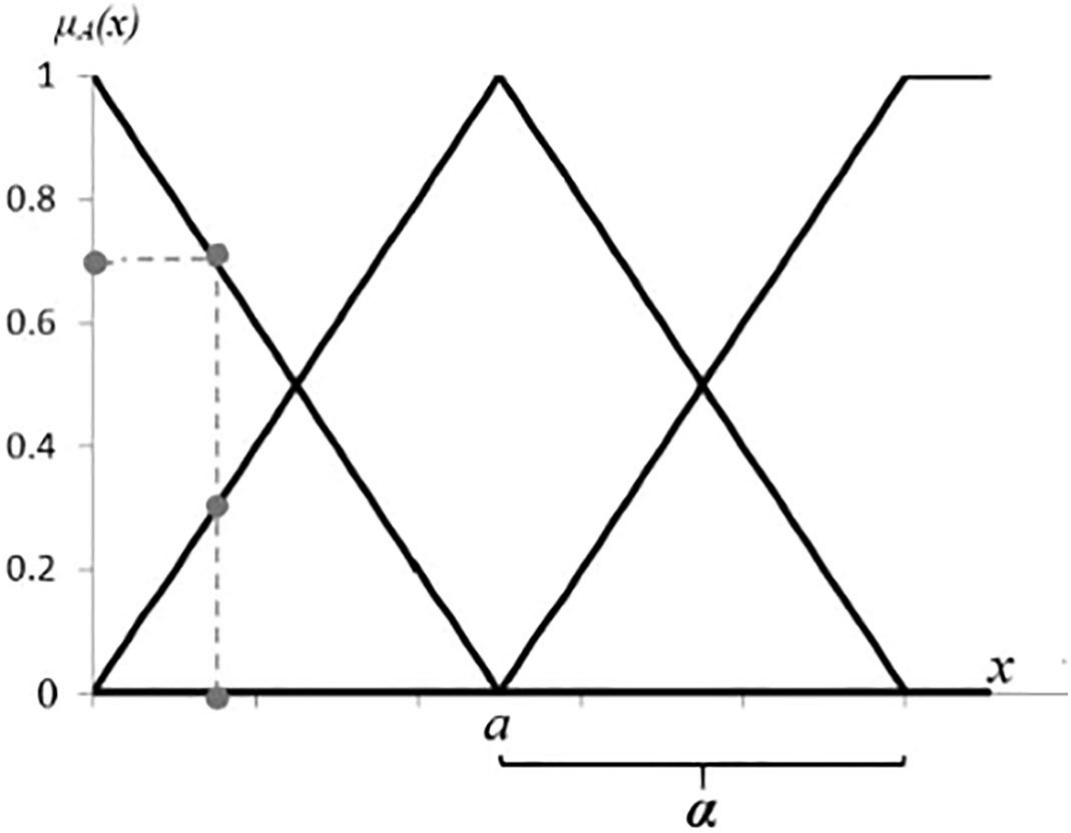
Symmetric triangular membership function.

Symmetric triangular membership function is also specified by two parameters as Eq. (3) and Fig. 1.

3 $$\mu_{A} (x) = \left\{ {\begin{array}{*{20}l} {1 - \frac{x - a}{{\varvec{\alpha}}}} \hfill & {if\;\left| {x - a} \right| \le {\varvec{\alpha}}} \hfill \\ 0 \hfill & {otherwise.} \hfill \\ \end{array} } \right.$$

#### Rules evaluation

The fuzzified inputs are applied to the rule antecedents. As the fuzzy rule has multiple antecedents, the AND or OR fuzzy operation is used to obtain a single number which represents the result of the antecedent evaluation. In this paper, the AND is applied to evaluate the conjunction of the rule antecedents, which is defined as Eq. (4).

4 $$\mu_{A1 \cap A2} (x)\; = \;\min \left\{ {\mu_{A1} (x),\mu_{A2} (x)} \right\},$$

In other application, if the OR fuzzy operation applies the classical fuzzy operation union, which is defined as Eq. (5).

5 $$\mu_{A1 \cup A2} (x) = \max \left\{ {\mu_{A1} (x),\mu_{A2} (x)} \right\}.$$

Now the antecedent evaluation result can be used to the membership function of the consequent, which is commonly based on two methods: clipping and scaling. Clipping method is to cut the consequent membership function by the truth value of the rule antecedent, and scaling method is to adjust the original membership function of the rule consequent by multiplying all its membership degrees at the level of the antecedent truth. Clipping loses some information, but involving less complex and easier to defuzzify. And scaling preserves the original shape of the fuzzy set. For simplification and interpretation, the method clipping is adopted in this paper.

#### Aggregation of the rule outputs

The membership functions of all consequents clipped in the previous step are combined into a single fuzzy set.

#### Defuzzification

The centroid technique is a popular defuzzification method. It finds the centroid point representing the center of gravity (COG) of the aggregated fuzzy set *R*. A reasonable estimate can be obtained by centroid deffuzification method, which is defined as Eq. (6).

6 $$z = \frac{{\sum\limits_{j = 1}^{M} {\overline{z}_{{^{{^{j} }} }} \left( {\prod\limits_{i = 1}^{n} {\mu_{{A_{i}^{j} }} (x_{i} )\mu_{{B^{j} }} \left( {\overline{z}_{{^{{^{j} }} }} } \right)} } \right)} }}{{\sum\limits_{j = 1}^{M} {\left( {\prod\limits_{i = 1}^{n} {\mu_{{A_{i}^{j} }} (x_{i} )\mu_{{B^{j} }} \left( {\overline{z}_{{^{{^{j} }} }} } \right)} } \right)} }},$$

where $\overline{z}_{{^{{^{j} }} }}$ is the point in *R* at which $\mu_{{B^{j} }} (z)$ achieves its maximum value (usually, we assume that $\mu_{{B^{j} }} \left( {\overline{z}_{{^{{^{j} }} }} } \right)$ = 1).

This method is a universal approximator, i.e. they can approximate any continuous function on a compact set to an arbitrary accuracy^17^.

### FCM

Clustering is an unsupervised learning method, which assigns a given set of objects into disjoint groups or clusters by membership degrees between 0 and 1. A high degree value represents a high similarity between the object and the group. FCM is a well-known fuzzy clustering algorithms^30^. The main motivation of proposing FCM was to address the deficiency in working with overlapping groups shown by the hard cluster algorithm. The FCM method was developed by researches recently^31–34^. In order to keep the simplicity and interpretability, the original FCM method is included in FCM-FN model. The process is expressed as follows^9^:

Let $\Omega = \{ 1, \ldots ,k, \ldots ,n\}$ be a set with *n* objects. Object *k* is the vector of quantitative variables $X_{k} = \{ x_{1k}, \ldots ,x_{jk}, \ldots ,x_{pk}\}$ described by *p* variables where $x_{jk} \in {\varvec{R}}$. Let $Y = \{ 1, \ldots ,i, \ldots ,c\}$ be a set of *c* prototypes associated to *c* groups, where each prototype *i* is a vector of quantitative variables $Y_{i} = \{ y_{1i}, \ldots ,y_{ji}, \ldots ,y_{pi}\}$, where $y_{ji} \in {\varvec{R}}$. Let $U = \left[ {u_{ik}} \right]$ be a *c* × *n* membership degree matrix, where ${\text{u}}_{ik}$ is the membership degree of object *k* to group *i*, where ${\text{u}}_{ik} \in [0,1]$. The algorithm works according to minimizing the objective function that is defined as Eq.(7). At the same time, a prototype matrix $Y^{*}$ and a membership degree matrix $U^{*}$ are obtained.

7 $$J(Y,U) = \sum\limits_{i = 1}^{c} {\sum\limits_{k = 1}^{n} {(u_{ik} )^{m} } d_{ik} ,}$$

where *m* is the value of cluster fuzziness, and $d_{ik}$ is the squared Euclidean distance which measures the dissimilarity of the feature vectors between $x_{k}$ and $y_{i}$. The distance is calculated by Eq. (8).

8 $$d_{ik} = \sum\limits_{j = 1}^{p} {\left( {x_{jk} - y_{ji} } \right)^{2} } .$$

Under the minimizing criterion *J*, the prototypes are updated according to Eq. (9), and the membership degrees are updated by Eq. (10) with the restriction $\sum\limits_{i = 1}^{c} {u_{ik} = 1}$.

9 $$y_{ji} = \frac{{\sum\limits_{k = 1}^{n} {(u_{ik} )^{m} x_{jk} } }}{{\sum\limits_{k = 1}^{n} {(u_{ik} )^{m} } }}.$$

10 $$u_{ik} = \left[ {\left. {\sum\limits_{a = 1}^{c} {\left( {\frac{{d_{ik} }}{{d_{ak} }}} \right)^{{\frac{1}{m - 1}}} } } \right]} \right.^{ - 1} .$$

### PSO

As mentioned in section “Introduction”, PSO is a typical optimization algorithm that searches for the best solution by modeling the social behavior of bird flocks and fish schools. The population of the birds is named “swarm”, and the members of the population are called “particles”. Assume that the Search space dimension is D, and the swarm is *Sw* = $\{ X_{1} , \ldots ,X_{s} \}$, where *s* is the total number of particles; A particle represents a position in D; the position of the *ith* particle in the search space is denoted as $X_{i} = \left( {x_{i1} , \ldots ,x_{id} , \ldots ,x_{iD} } \right),\;$ *i* = *1,2,…,s*. *pbest* records the position of *ith* particle’s previous optimal performance, which is denoted as $p_{i} = \left( {p_{i1} , \ldots ,p_{id} , \ldots ,p_{iD} } \right)$. *gbest* is the best position achieved by the swarm, which is expressed as $p_{g} = \left( {p_{g1} , \ldots ,p_{gd} , \ldots ,p_{gD} } \right)$. The velocity of the ith particle is denoted as $v_{i} = \left( {v_{i1} , \ldots ,v_{id} , \ldots ,v_{iD} } \right)$. *pbest* and *gbest* direct a particle’s new velocity and position. $p_{id}^{{^{{}} }} (t)$ is the *i*th particle’s optimal position, and $p_{gd}^{{}} (t)$ is the global best position of the *d*th dimension at instant *t*. The particles fly through the solution space towards better positions, and the process is implemented by Eqs. (11) and (12).

11 $$\begin{aligned} v_{id} (t + 1) & = \omega (t) \times v_{id} (t) + (c_{1} r_{1} ) \times \left( {p_{id} (t) - x_{id} {\text{(t)}}} \right) \\ & \;\; + (c_{2} r_{2} ) \times \left( {p_{gd} (t) - x_{id} (t} \right)), \\ \end{aligned}$$

12 $$x_{id} (t + 1) = x_{id} {\text{(t) + }}v_{id} (t + 1),$$

where the positive constants $c_{1}$ and $c_{2}$ are acceleration coefficients, *r*_*1*_ and *r*_*2*_ are random values in range [0,1]. $x_{id} {\text{(t)}}$ is the position and $v_{id} (t)$ is the velocity of *i*th particle in *d*th dimension at instant *t*. The inertia value $\omega (t)$ is obtained by Eq. (13).

13 $$\omega (t) = \omega_{\max } - \left( {\omega_{\max } - \omega_{\min } } \right) \times t/t_{\max } ,$$

where *t* is the current instant and $t_{\max }$ is the maximum iteration number. When iteration proceeds, the weighting factor of updating speed will decrease from the maximum factor $\omega_{\max }$ to the minimum one $\omega_{\min }$^11,15^.

To solve clustering problems, each particle represents a feasible solution to the optimization problem. Let *f* is an object function, and the personal best position of a particle at instant *t* is updated by Eq. (14).

14 $$p_{i} (t + 1) = \left\{ {\begin{array}{*{20}l} {p_{i} (t)} \hfill & {if\;f(X_{i} (t + 1)) \ge f(p_{i} (t))} \hfill \\ {X_{i} (t + 1)} \hfill & {if\;f(X_{i} (t + 1)) < f(p_{i} (t))} \hfill \\ \end{array} } \right.\;\;1 \le i \le S,$$

where $p_{g}^{{}} (t + 1)$ is the global best position at instant *t*+*1* found by anyone of all particles during the previous steps, defined by Eq. (15).

15 $$p_{g} (t + 1) = \arg \;\mathop {\min }\limits_{pi} \;f(p_{i} (t + 1))\;\;1 \le i \le S.$$

### PSO based tuning membership functions

In the proposed model, there are two kind of membership functions: Gaussian or triangular. PSO algorithm is specified to adjust the shapes of membership functions by tuning the parameters of membership functions.

The first tuning is aiming towards the parameters of Gaussian membership functions, which are generated by FCM. This process is abbreviated as FCM-PSO. The steps are described by Algorithm FCM-PSO(*Ω, c*), which returns matrix ***Y*** including two kinds of parameters(*θ* and *σ*) in Gaussian membership functions, as shown in Eq. (2).

The second tuning is aiming towards the parameters of symmetric triangular membership functions, which is specified by experts. This process, similar to FCM-PSO, returns matrix ***M*** including two kinds of parameters(*a* and α) in symmetric triangular membership functions, as shown in Eq. (3) and Fig. 1.
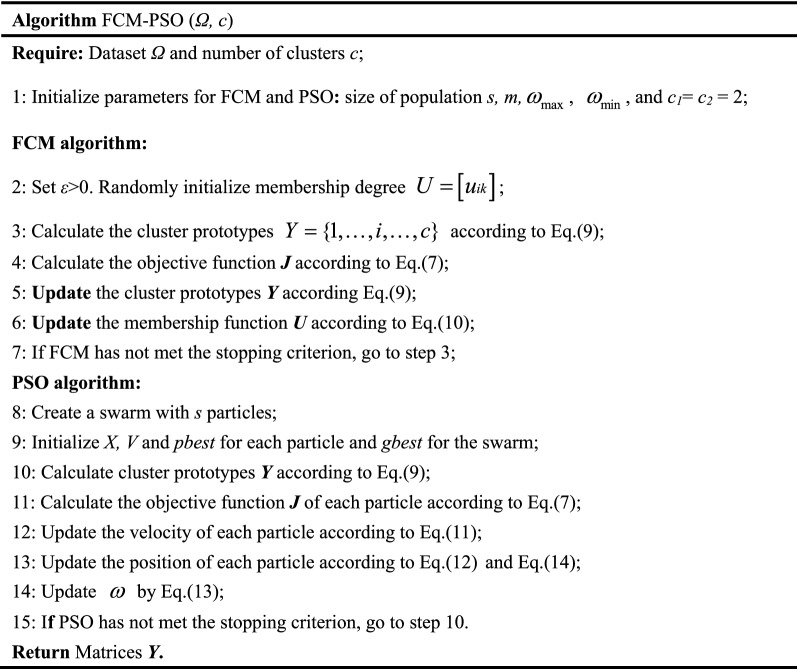


### FN

In this section, the details of the novel theory of FN are expressed, which include the basic theory, the basic operations and three important theorems. All the selected details are involved in the proposed model.

#### Basic theory of FN

Standard Fuzzy System (SFS) is the most known type of fuzzy system which is with a single rule base. SFS is characterized by the nature of a black-box where the outputs map the inputs directly, and internal connections are out of consideration^35,36^. Reflecting the influence of all inputs on the output simultaneously, SFS is quite accurate for output modeling usually. But, when the rules of SFS increasing, the transparency deteriorates, and it is less clear how the inputs affect the output.

Chained Fuzzy System (CFS), is with multiple rule bases in cascade, and characterized by the nature of white-box where the outputs map the inputs by connected internal variables^37,38^. CFS is with an arbitrary structure with the form of connected subsystems. It is applied as a detailed presentation of SFS for improving transparency by explicitly accounting all subsystems’ interactions. And the smaller number of inputs improve the efficiency of CFS. However, because of the error accumulation from the multiple Fuzzification-Inference-Defuzzification (FID) sequences, accuracy may be lost. Hierarchical Fuzzy System (HFS), a special kind of CFS, each subsystem in HFS only with one output and two inputs.

FN, a novel concept with networked rule bases^18,22,24^, is characterized by the nature of a white-box where the outputs map the inputs by connections. Arguably, FN is a hybrid of SFS and HFS. On one hand, the structure is similar between FN and HFS because of the explicit presentation of the interactions and subsystems. On the other hand, after the multiple-rule-bases of FN been simplified to a linguistically equivalent single rule base, the operation of FN is similar to SFS. The process of simplification implements the linguistic composition approach, including the vertical merging and horizontal merging of rule bases in FN. The multiple rule base system in HFS is converted into a FN, and then a FN is composed into a single-rule-base system.

As a hybrid concept, FN obtains the advantages of both accuracy from SFS and transparency from CFS/HFS. The structure’s transparency is directly related to interpretation of a prediction model, which has turned out to be same important than accuracy and efficiency for complex systems modelling^24^, and it is the core reason that FN is employed in the fuzzy model for prediction in this paper.

#### Basic operations of FN

Inputs and outputs of a FN model take linguistic terms. In this paper, the FN model is with only one output(MISO) and the If–then rules are in form of Eq. (1). For compactness, the linguistic terms of the inputs $\left( {\left\{ {A_{1}^{j} , \ldots ,A_{n}^{j} } \right\}} \right)$ and the outputs $\left( {\left\{ {B^{j} } \right\}} \right)$ in Eq. (1) are represented by positive integers. For example, {‘small’, ‘average’, ‘big’} are encoded as positive integers {‘1’, ‘2’, ‘3’}.

**Figure 2:**
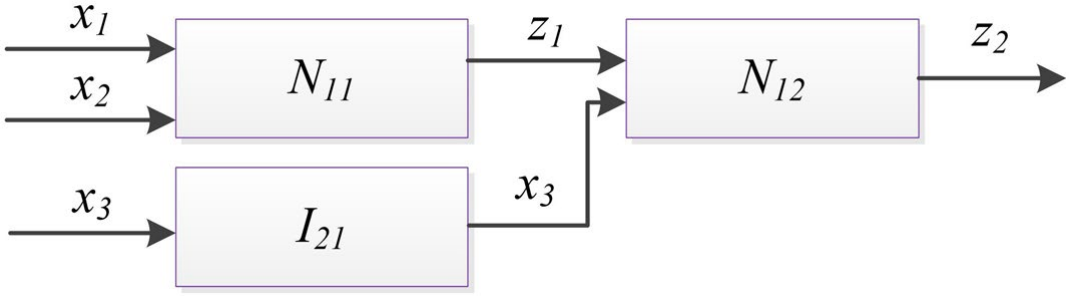
Hierarchical structure example for illustrating the basic operations in FN.

In order to illustrate the basic operations in FN, a simple example is shown as Fig. 2. A FN with 3 nodes *N*_*11*_*, N*_*12*_*, I*_*21*_ is described by the Boolean matrices given in Table 1. For simplicity, Each input (output) is only with two linguistic terms, represented by {‘1’, ‘2’}. More examples have been included in the folder “Mathlab functions” in the Supplementary File.

**Table 1:** Boolean matrices of nodes *N*_*11*_*, N*_*12*_*, I*_*21*_*.*

*N*_*11*_*: z*_*1*_	*1*	*2*	*I*_*21:*_* x*_*3*_	*1*	*2*	*N*_*12*_*: z*_*2*_	*1*	*2*
*x*_*1*_* x*_*2*_			*x*_*3*_			*z*_*1*_* x*_*3*_		
1 1	*1*	*0*	1	*1*	*0*	1 1	*1*	*0*
1 2	*1*	*0*	2	*0*	*1*	1 2	*1*	*0*
2 1	*1*	*0*				2 1	*0*	*1*
2 2	*0*	*1*				2 2	*0*	*1*

The rules of *N*_*11*_ are same to the if–then rules given in Eq. (16). The node of *N*_*12*_ is similar to *N*_*11*_.

16 $$\begin{gathered} 
{\text{if }}\;{\text{x}}_{{1}} \, \;{\text{is }}\;{1 }\;{\text{and }}\;{\text{x}}_{{2}} \, \;{\text{is }}\;{1}\;{\text{ then}}\;{\text{ z}}_{{1}} \;{\text{ is}}\;{ 1} 
\hfill \\ 
{\text{if }}\;{\text{x}}_{{1}} \, \;{\text{is }}\;{1 }\;{\text{and}}\;{\text{ x}}_{{2}} \, \;{\text{is }}\;{2}\;{\text{ then}}\;{\text{ z}}_{{1}} \;{\text{ is}}\;{ 1} 
\hfill \\ 
{\text{if }}\;{\text{x}}_{{1}} \, \;{\text{is }}\;{2 }\;{\text{and}}\;{\text{ x}}_{{2}} \, \;{\text{ is }}\;{1 }\;{\text{then }}\;{\text{z}}_{{1}} \;{\text{ is}}\;{ 1} 
\hfill \\ 
{\text{if }}\;{\text{x}}_{{1}} \, \;{\text{is }}\;{2 }\;{\text{and }}\;{\text{x}}_{{2}} \, \;{\text{ is}}\;{ 2}\;{\text{ then }}\;{\text{z}}_{{1}} \;{\text{ is}}\;{ 2} \hfill \\ 
\end{gathered}$$

The special node of *I*_*21*_ is an identity node, and the outputs is same to the inputs. The rules of *I*_*21*_ is described by if–then rules given in Eq. (17).

17 $$\begin{gathered} 
{\text{if }}\;{\text{x}}_{{3}} \;{\text{ is}}\;{ 1}\;{\text{ then }}\;{\text{x}}_{{3}} \;{\text{ is}}\;{ 1} 
\hfill \\ 
{\text{if }}\;{\text{x}}_{{3}} \;{\text{ is}}\;{ 2}\;{\text{ then }}\;{\text{x}}_{{3}} \;{\text{ is}}\;{ 2} \hfill \\ \end{gathered}$$

Vertical merging is a kind of binary operation that can be applied to a pair of parallel nodes, i.e. nodes locate in the same layer of a FN. In Fig. 2, the nodes *N*_*11*_ and *I*_*21*_ are in the same layer. The vertical merging operation is identical with Boolean matrix Kroneker product. This kind of operation merges the operand nodes from the pair into a single product node. In this case, the inputs to the product node represent the union of the inputs to the operand nodes where the outputs from the product node represent the union of the outputs from the operand nodes. The operation of vertical merging can always be applied due to the ability to concatenate the inputs and the outputs of any two parallel nodes^24^. The symbol ‘+’ denotes the vertical merging operation.

**Figure 3:**
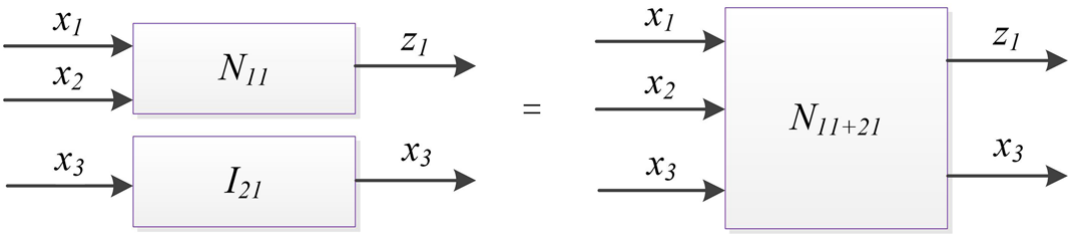
Block-scheme of two nodes’ vertical merging into one node with 3 inputs and 2 outputs.

In Fig. 3, nodes *N*_*11*_ and *I*_*21*_ represent a two-node subnetwork of this FN. This two-node FN can be described by the block-scheme and the topological expression in Eq. (18), The vertical merging of the operand nodes *N*_*11*_ and *I*_*21*_ results into a single product node *N*_*11*+*21*_ which represents a simplified image of the two-node FN in the form of a one-node FN. Due to the concatenation of the inputs to the operand nodes as inputs *x*_*1*_, *x*_*2*_ and *x*_*3*_ to the product node and the concatenation of the outputs from the operand nodes as outputs *z*_1_ *and x*_*3*_ from the product node. This node *N*_*11*+*21*_ can be described by the topological expression in Eq. (19) and the Boolean matrix in Table 2.

18 $$\left[ {N_{11} } \right](x_{1} ,x_{2} |z_{1} ) + \left[ {I_{21} } \right](x_{3} |x_{3} ),$$

19 $$\left[ {N_{11 + 21} } \right](x_{1} ,x_{2} ,x_{3} |z_{1} ,x_{3} ).$$

**Table 2:** Boolean matrix of node *N*_*11*+*21*_*.*

*N*_*11*+*21*_*: z*_*1*_*, x*_*3*_	*1 1*	*1 2*	*2 1*	*2 2*
*x*_*1*_* x*_*2*_* x*_*3*_				
1 1 1	*1*	*0*	*0*	*0*
1 1 2	*0*	*1*	*0*	*0*
1 2 1	*1*	*0*	*0*	*0*
1 2 2	*0*	*1*	*0*	*0*
2 1 1	*1*	*0*	*0*	*0*
2 1 2	*0*	*1*	*0*	*0*
2 2 1	*0*	*0*	*1*	*0*
2 2 2	*0*	*0*	*0*	*1*

Each node in Eqs. (18) and (19) is placed in a pair of square brackets ‘[ ]’. The inputs and the outputs for each node are placed in a pair of simple brackets ‘( )’ right after the node, where the outputs are separated from the inputs by a vertical slash ‘|’. The topological expressions in the rest of this paper are with the similar structure.

Horizontal merging is a binary operation that can be applied to a pair of sequential nodes, i.e. nodes located in the same level of a FN. This operation merges the operand nodes from the pair into a single product node. The product node has the same input as the input to the first operand node and the same output as the output from the second operand node whereas the connection does not appear in the product node.

**Figure 4:**
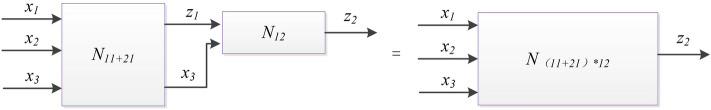
Block-scheme of two nodes’ horizontal merging into one node with 3 inputs and 1 outputs.

As described by the block-scheme in Fig. 4, the two-node FN can be expressed by the topological Eq.(20). The symbol ‘*’ implies that the operation of horizontal merging. The horizontal merging of the operand nodes *N*_*11*+*21*_ and *N*_*12*_ results into a single product node *N*_*(11*+21)*12_. As a simplified image of the two-node FN in the form of one-node, *N*_*(11*+21)*12_ is descried by the topological expression in Eq. (21) and the Boolean Matrix in Table 3.

20 $$\left[ {N_{21 + 21} } \right](x_{1} ,x_{2} ,x_{3} |z_{1} ,x_{3} )*\left[ {N_{22} } \right](z_{1} ,x_{3} |z_{2} ),$$

21 $$\left[ {N_{(11 + 21)*12} } \right](x_{1} ,x_{2} ,x_{3} |z_{2} ).$$

**Table 3:** Boolean Matrix of *N*_*(11*+21)*12_*.*

*N*_*(11*+21)*12_*: z*_*2*_	*1*	*2*
*x*_*1*_* x*_*2*_* x*_*3*_		
1 1 1	*1*	*0*
1 1 2	*1*	*0*
1 2 1	*1*	*0*
1 2 2	*1*	*0*
2 1 1	*1*	*0*
2 1 2	*1*	*0*
2 2 1	*0*	*1*
2 2 2	*0*	*1*

#### Three important theorems

The three theorems are the important theoretical base for the proposed model in this paper^22^.

##### Theorem 1.

Denoted by the symbol ‘+’, the vertical merging operation is associative according to Eq. (22).

22 $$(M + N) + {\text{O}} = M + (N + O),$$

where for any three operand nodes *M, N, O,* the vertical merging from bottom to top is equal to their vertical merging from top to bottom.

##### Theorem 2.

Denoted by the symbol ‘∗’, the operation of horizontal merging is associative according to Eq. (23).

23 $$(M*N)*O = M*(N*O),$$

where for any three operand nodes *M, N, O,* the vertical merging from right to left is equal to their vertical merging from left to right.

##### Theorem 3.

**Figure 5:**
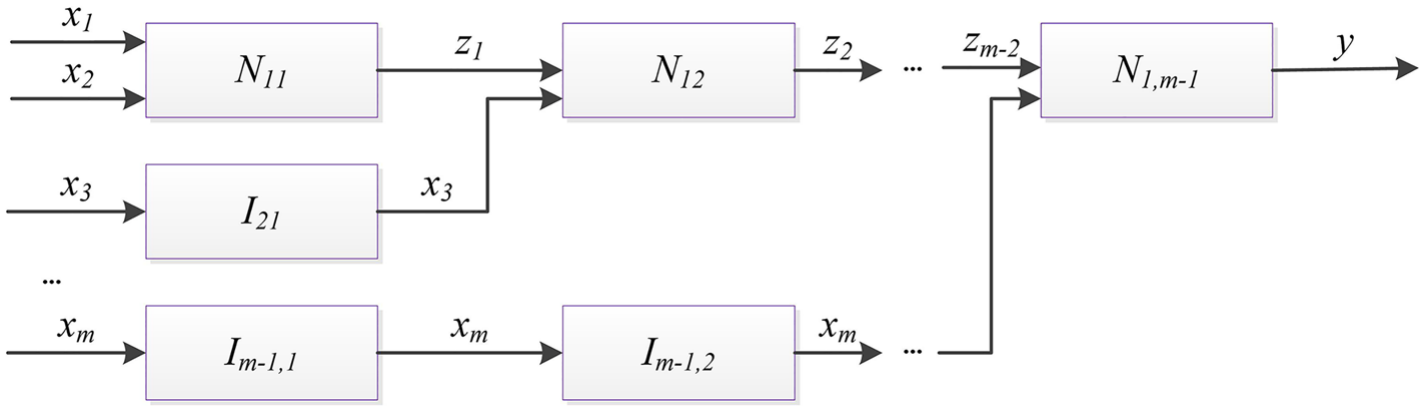
Block-scheme of Fuzzy Networks for theorem expression.

A HFS with inputs $\{ x_{1} ,x_{2} , \ldots ,x_{{\text{m}}} \}$, network nodes $\{ N_{11} ,N_{12} , \ldots ,N_{{\text{1,m - 1}}} \}$, connections $\{ z_{1} ,z_{2} , \ldots ,z_{{\text{m - 2}}} \}$ and a output y, as described by the topological expression in Eq. (24)

24 $$[N_{11} ](x_{1} ,x_{2} |z_{1} )*[N_{12} ]\left( {z_{1} ,x_{3} |z_{2} } \right)* \ldots *[N_{1,m - 1} ]\left( {z_{m - 2} ,x_{m} |y} \right)$$

can be characterized as a SFS with the same set of *m* inputs, the same single output, a single network node *N*, and no connections as described by the block-schemes in Fig. 5 and the topological expression in Eq. (25).

25 $$\left[ {\mathop \prod \limits_{p = 1}^{m - 1} \left( {N_{1p} + \mathop \sum \limits_{q = p + 1}^{m - 1} I_{qp} } \right)} \right]\left( {x_{1} ,x_{2} , \ldots ,x_{m} {\text{|y}}} \right),$$

where ${ }N = \mathop \prod \limits_{p = 1}^{m - 1} \left( {N_{1p} + \mathop \sum \limits_{q = p + 1}^{m - 1} I_{pq} } \right)$, node *I*_*qp*_ is identity node in level *p* and layer *q*.

## The proposed fuzzy model for prediction

**Figure 6:**
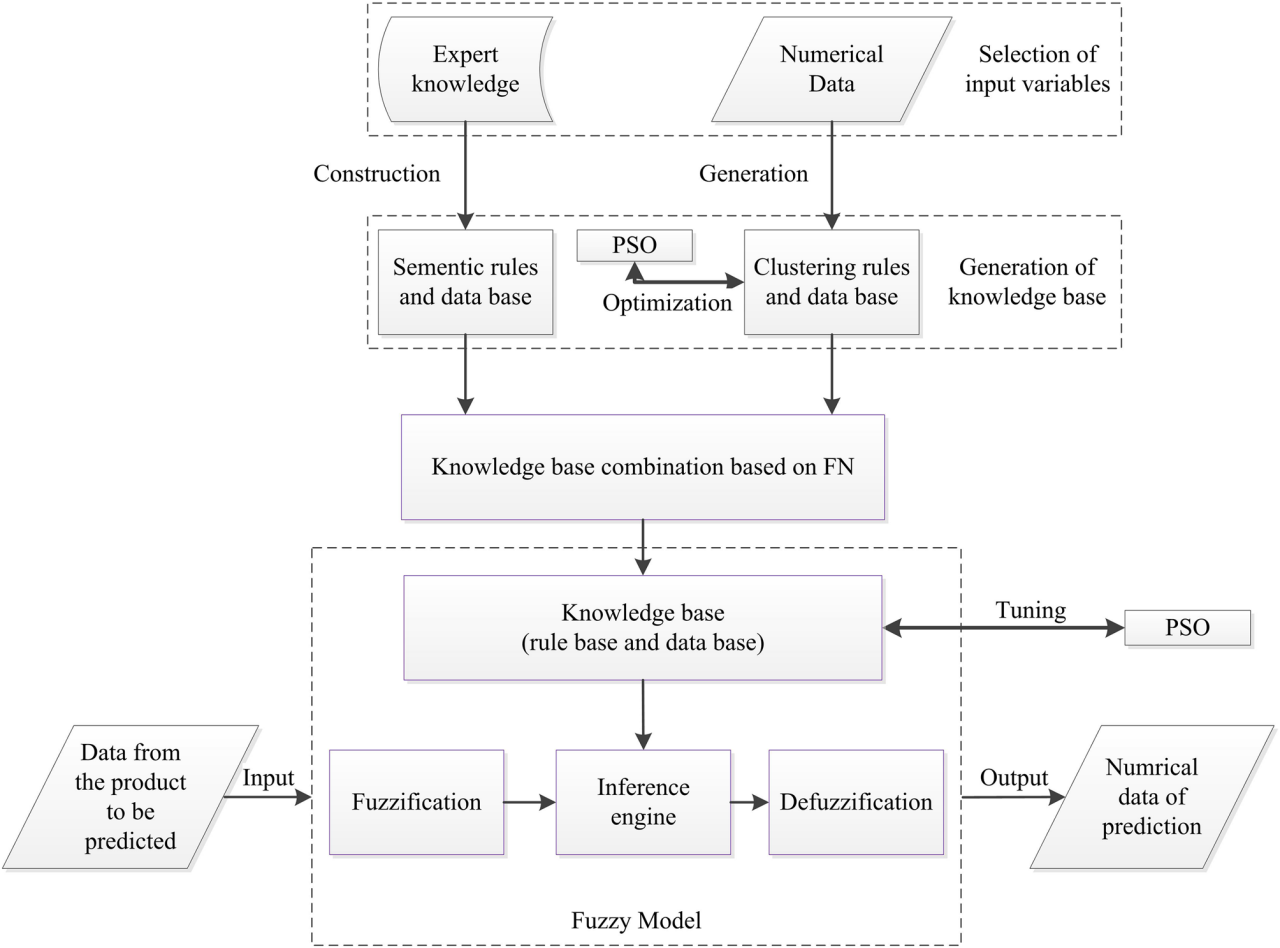
Flow sheet of construction the FCM-FN model.

**Figure 7:**
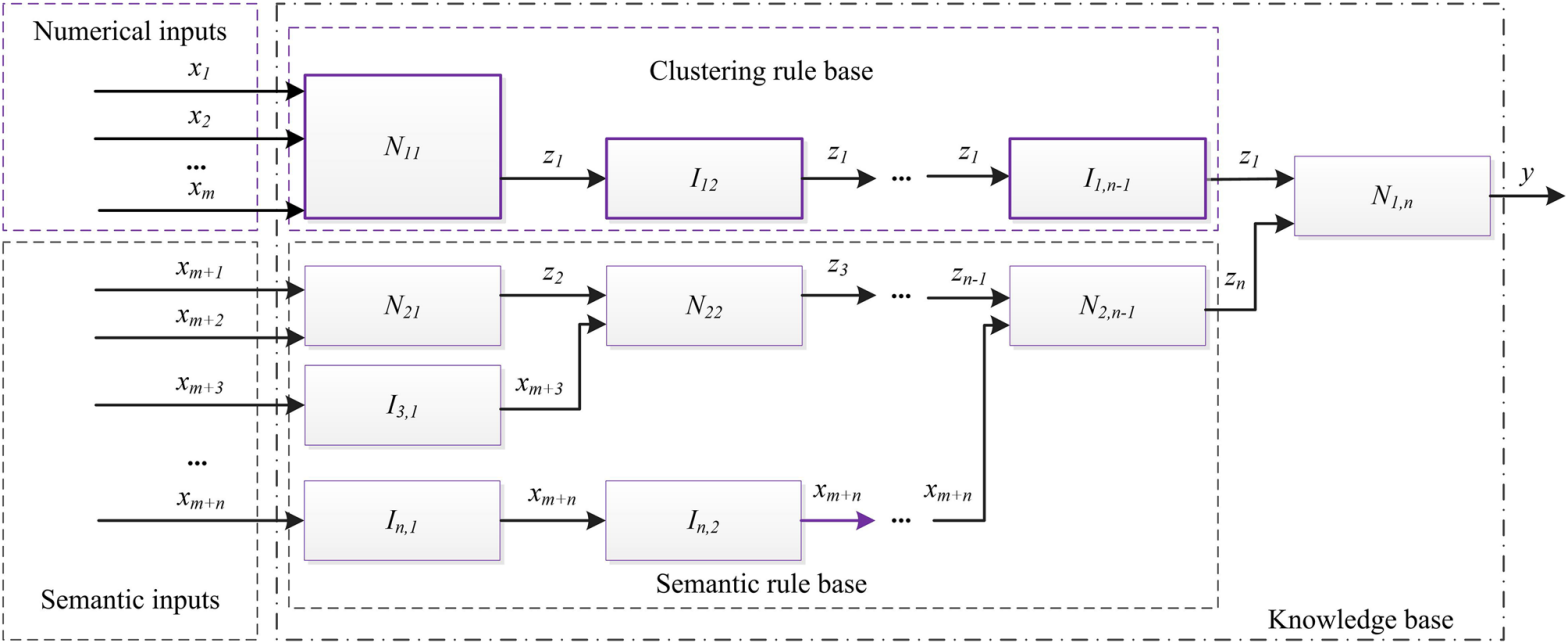
FN structure of the proposed model.

This section describes the proposed FCM-FN model, the process of construction the model includes 4 steps, as shown in Fig. 6. The first step aims at selection of input variables, based on the available numerical data and expert knowledge. The second step aims towards generation of knowledge base. The semantic rules and their data bases (DB1) are constructed from expert knowledge, and the clustering rules and its data base (DB2) are generated from numerical data by FCM algorithm. Then, the parameters of DB2 are optimized by the PSO algorithm. In the third step, the two kind of rule bases in second step are combined based on FN method. The forth step aims to optimize the parameters of DB1 also based on PSO algorithm. After the four steps, the proposed model as Fig. 7 is converted into a SFS.

Being converted into a SFS, the proposed FCM-FN model is a MISO system consisting of four major modules: fuzzification, inference engine, defuzzification and knowledge base. The fuzzification module converts the crisp inputs of independent variables to linguistic terms. And these linguistic terms combined with the ones from experts are processed in fuzzy domain by inference engine based on FN. The knowledge base is composed of the rule base, characterizing the control goals and control policy by a set of rules; and of the data base, containing the linguistic term sets and the membership functions defining their semantics. Finally, the processed output is transformed from fuzzy domain (linguistic terms) to crisp domain (numerical number) by defuzzification module^18^.

### Selection of input variables

There is large amount of indices to character a product. To reduce the complexity of a model, only those metrics and attributes with significant contribution to the prediction are selected. In practices, experts always are with the knowledge that some metrics are more important than others. Pearson Correlation Coefficient ($r_{p}$, expressed as Eq. (26)) and Spearman Correlation Coefficient ($r_{r}$, expressed as Eq. (27)) indicate the strength and direction of a linear relationship between two variables, which are useful to get top relevant input variables.

26 $$r_{p} = \frac{{\sum\limits_{i = 1}^{n} {\left( {x_{i} - \overline{x} } \right)\left( {y_{i} - \overline{y} } \right)} }}{{\sqrt {\sum\limits_{i = 1}^{n} {\left( {x_{i} - \overline{x} } \right)^{2} } } \sqrt {\sum\limits_{i = 1}^{n} {\left( {y_{i} - \overline{y} } \right)^{2} } } }},$$

where *x* is values in the first set of data, *y* is values in the second set of data, *n* is total number of values.

27 $$r_{r} = 1 - \frac{{6\sum {d_{i}^{2} } }}{{n\left( {n^{2} - 1} \right)}},$$

where $d_{i}^{2}$ is the difference between the *x* rank and the *y* rank for each pair of data, $\sum {d_{i}^{2} }$ is sum of the squared differences between *x* and *y* variable ranks, *n* is sample size.

As showed in Fig. 7, the selected metrics *x*_*i*_ (*i* = *1, 2,…, m* + *n*) are input variables. there are *m* + *n* inputs divided into two sets. SET1 is composed of numerical inputs *x*_*i*_ (*i* = *1, 2,…, m*) which are from the variables with numerical data, and SET2 is composed of semantic inputs *x*_*i*_ (*i* = *m* + *1, m* + *2, …, m* + *n*) from the variables only with the expert’s linguistic information. Combined with the numerical data of output *z*_*1*_ (here *z*_*1*_ is the prediction variable), the numerical data are transformed into a set of input–output data pairs.

### Generation and optimization of knowledge base

The process of generation and optimization of knowledge base includes three parts: generation of rule base, generation of data base and parameters optimization of Gaussian membership functions.

#### Generation of rule base

Given a set of input–output data pairs as Eq. (28), the sampling data is preprocessed, including deleting the duplicated data pairs and min–max normalization according to Eq. (29).

28 $$\left( {x^{(p)} ; \, y^{(p)} } \right), \, p = 1,2, \ldots ,N,$$

where $$x^{(p)} \in {\text{R}}^{m} \;and\;y^{(p)} \in {\text{R}}$$.

29 $$x^{\prime} = \frac{{x - x_{\min } }}{{x_{\max } - x_{\min } }},$$

where $x^{\prime}$ is the min–max normalized set, *x* is a set of the observed values present in *x*, $x_{\min }$ is the minimum values in *x,* $x_{\max }$ is the maximum values in *x.*

The basic problem is to extract rules which describe how the output variable $y \in {\text{R}}$ is influenced by the *m* input variables $x = (x_{1} , \ldots ,x_{m} )^{T} \in R^{m}$ based on Eq. (28). FCM is a powerful unsupervised learning technique to extract rules of Mamdani structure as Eq. (1), which divides sampling data into several clusters based on their similarity^5^. In the proposed model, *K* is the number of clusters and specified to be a positive odd integer. After the sampling data is clustered by FCM algorithm, the input space of each numerical input is divided into *K* sections, and *K* Mamdani rules is generated. The rule base of Node *N*_*11*_ in Fig. 7 is composed by these *K* rules.

The semantic inputs in SET2 and outputs are Subjective Product Appraisals (SPA). For simplicity and interpretability, the input space of these semantic inputs and outputs are also divided into *K* sections. For example, if *K* = 3, the semantic choice scale is {Low, Nominal, High} presented by the positive integers 1–3. These rule bases are derived from the knowledge of experts and engineers. It is worth noting that the quality of the rule bases affects the output to some degree. The rule base for Nodes *N*_*1n*_ and *N*_*2j*_ (*j* = *1,…,n−1*), which are associated with these semantic inputs, is Mamdani type as Eq. (1), and the number of rules is not over *K*^2^ because of only two inputs of each node.

The identity nodes *I*_*ij*_ (*i* = *1,…,n; j* = *1,…,n−1*) are with the rule base of Mamdani type, too. And the number of rules is *K* because of only one input of each identity node.

As shown in Eq. (1), each fuzzy rule has multiple antecedents. The AND fuzzy operation intersection is implemented, which is defined as Eq. (4). The reason is that all the input variables simultaneously affect the output, and rules in antecedent must be met simultaneously in order for consequent to occur. For prediction problems, only “and” rules are required since the antecedents are different components of a single input vector.

#### Generation of data base

The data base is associated with the type of membership functions. The process of assignment membership functions can be intuitive or based on some algorithmic operations. Six straightforward methods are described in the literature to assign membership functions to fuzzy variables. Based on the judgment about the probability density functions from researchers and ourselves, the shape of membership function of input variables of node *N*_*11*_ is Gaussian curve, as defined in Eq. (2). The position of the peak relative to the universe and the standard deviation are two parameters of a Gaussian membership function. The parameters are determined by FCM algorithm based on sampling data. In this paper, the result of FCM algorithm is named as FCM model, in which the knowledge base is with parameters of Gaussian membership functions.

For these semantic inputs, a natural membership function that readily comes to mind is symmetric triangular membership functions defined as Eq. (3). Two main reasons motivate the choice: one is their optimal interface design and the other is its semantic integrity.

#### Parameters optimization of Gaussian membership functions

In this step, the optimization is aimed towards the data base of the FCM model and based on the sampling data as Eq. (3). MATLAB-Fuzzy Logic Tool Box (**genfis**) is utilized to generate the FIS of FCM model. To improve the precision as well as reduce the loss of the interpretability, the optimization is only aiming towards parameters (*θ* and σ) of inputs and the output. The structure of this FIS is unchanged. As mentioned in section “PSO”, as a global optimization method, PSO algorithm is suitable for initial training to tune the parameters. In the proposed model, the MATLAB function “**tunefis**” is employed with the specified tuning algorithm name as “**particleswarm**”. The optimized model is named as FCM–PSO in this paper. It works by running the FCM method until it reaches its stopping criterion. Then it runs the PSO algorithm to try to achieve a better solution. In the experiments presented in section “Case study”, the following stopping criteria were used:

FCM: when there is a variation less than or equal to 1e−8 on Minimum improvement in objective function (MinImprovement for short).

PSO: when relative change in the best objective function value is less than 1e-8 on FunctionTolerance (a method option of “**tunefis**”) or it reaches maximum number of iterations (MaxNumIteration for short). The experiments explored 11 different MaxNumIterations in the set {10, 20, 30, 40, 50, 100, 200, 300, 400, 1000, 2000}.

### FN based knowledge base combination

The semantic inputs are organized into HFS structure as shown in Fig. 7. Two input variables (*x*_*m*+*1*_ and *x*_*m*+*2*_) are organized into *N*_*21*_, and other input variables are added one by one after converted by the identify node. And *N*_*2,n−1*_ combines all *n* semantic inputs. Identify nodes *I*_*1j*_ (*j* = *2,…,n−1*) are employed to combine *N*_*2,n−1*_ with *N*_*11*_. *N*_*1n*_ is the final node which combine numerical inputs with semantic inputs.

To combine these FN nodes into a SFS, the rule bases of all nodes are merged vertically and horizontally^39^. The rule bases of *N*_*11*_*, N*_*21*_*, I*_*31*_*,* … and *I*_*n,1*_ are merged firstly, the result named rule base *V*_*1*_, and in the same method, rule base *V*_*2*_ is from *I*_*12*_*, N*_*22*_*,* … and *I*_*n,2*_, rule base *V*_*n−1*_ is from the last vertical merging of *I*_*1,n−1*_ and *N*_*2,n−1*_. And then, all the rule base of *V*_*i*_* (0* < *i* ≤ *n−1)* are merged horizontally. Till now, the single rule base RB of the proposed model is generated, as topological expression in Eq. (30).

30 $$\begin{gathered} \left[ {\left( {N_{11} + N_{21} + I_{31} + \cdots + I_{n,1} } \right)} \right.*\left( {I_{12} + N_{22} + \cdots + I_{n - 1,2} + I_{n,2} } \right)* \hfill \\ \;\;\; \ldots \left( {I_{1,n - 2} + N_{2,n - 2} + I_{n,n - 2} } \right)*\left( {I_{1,n - 1} + N_{2,n - 1} } \right)*\left. {N_{1,n} } \right]\left( {x_{1} , \ldots ,x_{m + n} |y} \right), \hfill \\ \end{gathered}$$

where “ + ” means vertical merging operation and “ ∗ ” means horizontal merging operation.

**Figure 8:**
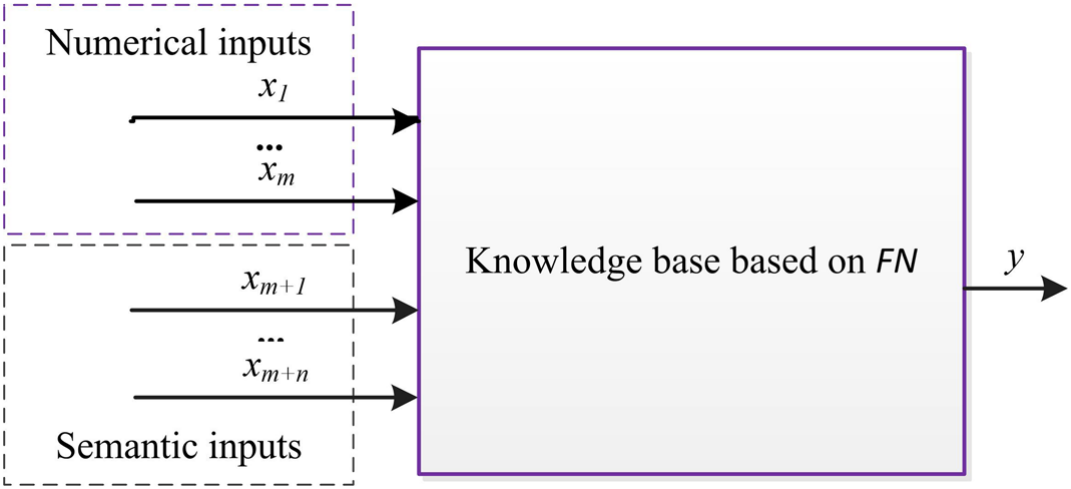
SFS structure after knowledge base combination based on FN method.

After the multiple rule bases are merged into a linguistically equivalent single rule base, the FN model is converted to a kind of SFS model with *m* + *n* inputs, a output and a knowledge base RB, as shown in Fig. 8.

### Tuning parameters of triangular membership functions

In this step, the optimization is implemented to the data base of *N*_*2j*_ (*j* = *1,…,n−1*) in Fig. 7. The initial values of parameters (*a* and *α*) of symmetric triangular membership functions, which was specified by experts, are tuned by PSO algorithm.

To implement the tuning, SPAs of *N*_*2j*_ should be transformed to numerical data. For example, the Eq. (31) can finish this transform. The semantic appraisal for each semantic input is from the experts’ judgments.

31 $${\text{D}}_{SPAi} = \frac{i - 1}{{{\text{K}} - 1}}\;\;\;\;\left( {1 \le {\text{i}} \le {\text{K}}} \right),$$

where *i* is the *i*th item in the linguistic term set. *K* is the number of items of the linguistic term set.

**Figure 9:**
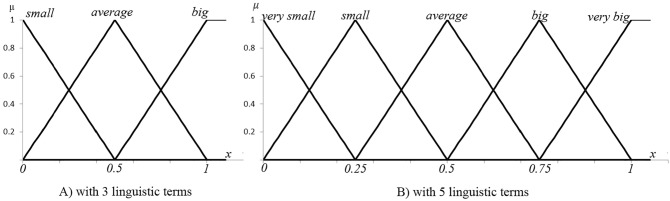
Symmetric triangular membership function with 3 linguistic terms and 5 linguistic terms respectively.

For example, the linguistic term set {small, average, big} have 3 items, in which the item “average” can be converted to 0.5, as shown in Fig. 9A. And {very small, small, average, big, very big} have 5 items, and the item “big” is same to 0.75, as shown in Fig. 9B. What’s more, the experts can appraise a semantic input by using a real number in the interval [0, 1] directly with the help of membership functions.

Combining ${\text{D}}_{SPAi}$ of *x*_*m*+*1*_* to x*_*m*+*n*_ with data pairs(*x*_*1*_ to *x*_*m*_*,* and *y*) produces new data pairs for FN. Two different type of input data are included in the new data pairs. One is from numerical inputs and this kind of inputs generally is quantifiable. For example, Lines of a paragraph of software code can be considered as numerical input. The other is from qualitative inputs. For example, programming language complexity is always appraised by semantic terms.

The new data pairs for FN is utilized to tune the parameters of triangular membership functions. The process of optimization is similar to section “Parameters optimization of Gaussian membership functions”, and with the same stopping criteria.

After being optimized in this step, the knowledge base RB of the proposed model is renewed and ready for prediction.

### Derivation of prediction value

The proposed model with optimized knowledge base has been converted into a SFS model as shown in Fig. 8 in the previous steps. Given a data pairs of *m* numerical inputs and *n* semantic inputs, the prediction value is yielded according to the FCM-FN model.

## Case study

In this section, a simple FCM-FN model was applied to 6 real world datasets to evaluate the performances. The details are described in 4 parts: the simple FCM-FN model, the performance metrics, the 6 datasets, and the results of case study. The model was implemented on a computer with an Intel Core i7–9750, 2.60 GHz processor and 16 GB RAM, running a Windows 10(64-bit) operating system and MATLAB R2019a.

### Simple FCM-FN model

Prior to building the rules, the number of linguistic variables is limited to a considerable size to avoid excessive rule explosion and deteriorated interpretation. This is because potentially, every combination of variables could require a distinct rule. Due to this, the linguistic terms used in the performance evaluation are limited to 3 which are {poor, fair and good}, or {low, moderate and high}^40^.

**Figure 10:**
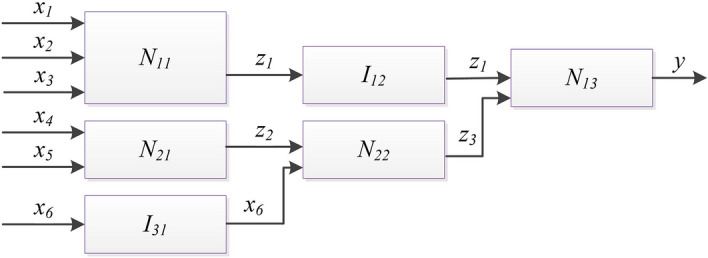
Simple FCM-FN model with 3 numerical inputs and 3 sematic inputs.

In order to show the interpretation and accuracy of the model, a simple FCM-FN model with only 3 numerical inputs and 3 semantic inputs is set up, as shown in Fig. 10. And 6 real world datasets are selected, which are descripted in section “Datasets”. For selection of the input variables *x*_*1*_ to *x*_*3*_ of each dataset, Pearson's and Spearman’s Correlation Coefficients are utilized to select top 3 relevant input variables according to the strength and direction of a linear relationship between each input and the output. The definitions of inputs *x*_*4*_ to *x*_*6*_ depend on the needs of prediction. The data pairs of all 6 inputs and a output is nondimensionalized according to Eq. (4).

In this simple FCM-FN model, the number of linguistic sets is specified to 3(*K* = 3). It means that the clusters in FCM is 3, *x*_*1*_ to *x*_*3*_, *z*_*1*_, *y* are with 3 Gaussian membership functions and *x*_*4*_ to *x*_*6*_, *z*_*2*_, *z*_*3*_ are with 3 symmetric triangular membership functions, separately.

Each node of *N*_*21*_, *N*_*22*_, *N*_*13*_ is with 9 (= 3^2^) rules. For example, in order to predict the software’s quality, the input variable *x*_*4*_ is specified as extent of the supporting document set, *x*_*5*_ is comprehensibility of the supporting document set and *x*_*6*_ is programming language complexity. They are organized into hierarchical structure, as shown in Fig. 10. The structure was not easy to make sense because *z*_*2*_ and z_3_ should be with exact connotation. Here, *z*_*2*_ is the subjective complexity appraisal, and z_3_ is subjective supporting environment appraisal. As shown in Table 4, the rule bases of *N*_*21*_*, N*_*22*_ and *N*_*13*_ are presented by Boolean matrices and derived by the knowledge of software engineer experts.

**Table 4:** Example of Rule bases of *N*_*21*_*, N*_*22*_ and *N*_*13*_*.*

*N*_*21*_*: z*_*2*_	*1*	*2*	*3*	*N*_*22*_*: z*_*3*_	*1*	*2*	*3*	*N*_*13*_*: y*	*1*	*2*	*3*
*x*_*4*_* x*_*5*_				*z*_*2*_* x*_*6*_				*z*_*1*_* z*_*3*_			
1 1	*1*	*0*	*0*	1 1	*1*	*0*	*0*	1 1	*1*	*0*	*0*
1 2	*0*	*1*	*0*	1 2	*1*	*0*	*0*	1 2	*1*	*0*	*0*
1 3	*0*	*1*	*0*	1 3	*1*	*0*	*0*	1 3	*1*	*0*	*0*
2 1	*0*	*1*	*0*	2 1	*0*	*1*	*0*	2 1	*0*	*1*	*0*
2 2	*0*	*0*	*1*	2 2	*0*	*1*	*0*	2 2	*0*	*1*	*0*
2 3	*0*	*0*	*1*	2 3	*0*	*0*	*1*	2 3	*0*	*0*	*1*
3 1	*0*	*0*	*1*	3 1	*0*	*0*	*1*	3 1	*0*	*0*	*1*
3 2	*0*	*0*	*1*	3 2	*0*	*0*	*1*	3 2	*0*	*0*	*1*
3 3	*0*	*0*	*1*	3 3	*0*	*0*	*1*	3 3	*0*	*0*	*1*

The knowledge base of *N*_*11*_ is generated by FCM algorithm, and the rule base includes 3 rules, and each identification node is also with 3 rules. Being merged based on FN method, the rule base of the simple FCM-FN model is with 81 rules.

### Performance metrics

As opposed to most existing approaches where the focus is to improve accuracy, the FCM-FN method focuses to maintain interpretation, transparency and accuracy by means of the modular rule bases that reflect the subsystems of the modeled system. There are three performance indicators to show the quality of the associated models. They are called Transparency Index (*TI*)^22^, Interpretation Index(*PI*) and Accuracy Index (*AI*).

The first performance indicator *TI* reflects the transparency according to the extent of its opaqueness from the inside of a model, as shown by Eq. (32).

32 $$TI = \frac{t + 1}{{p + q}},$$

where *t* is the total number of inputs, 1 is refer to the only one output, *p* is the number of non-identity nodes and *q* is the number of non-identity connections. A lower *TI* implies better transparency.

For example, in the simple FCM-FN model as shown in Fig. 10, *t* = 6, *p* = 4, *q* = 3,and *TI* = 1.

The second performance indicator *PI* reflects the interpretability of a model. It’s obvious that transparency is helpful to interpretability and more parameters impact the interpretability. Thus, we put forward a modified *TI* to reflects the interpretability of a FCM-FN model, as shown by Eq. (33).

33 $$PI = \frac{t + 1}{{p + q}}*\log_{10} (r + s + 10),$$

where *r* is the total number of parameters, *s* is the total number of optimized parameters. A lower *PI* implies better interpretability.

For example, in the model showed in Fig. 10, *t* = 6, *p* = 4, *q* = 3, *r* = 20, *s* = 14, and *PI* = 1.646.

The third performance indicator *AI* reflects the accuracy of a model by means of Symmetric Mean Absolute Percentage Error (*SMAPE*) as shown by Eq. (35) and Goodness-of-Fit (*R*^*2*^) as shown by Eq. (9).

34 $$SMAPE = \frac{100\% }{n}\sum\limits_{i = 1}^{n} {\frac{{\left| {prediction - actual} \right|}}{{\left( {\left| {prediction} \right| + \left| {actual} \right|} \right)/2}}} ,$$

35 $$R^{2} = 1 - \frac{{\sum\limits_{i = 1}^{n} {\left( {prediction - actua{\text{l}}} \right)^{2} } }}{{\sum\limits_{i = 1}^{n} {\left( {actual - mean} \right)^{2} } }},$$

where *n* is the total number of observations compared, *actual* is the *i*th value of the test dataset estimated values vector, and *prediction* is the *i*th value of output vector of a model, *mean* is the mean value of *actual*. Lower *SMAPE* or higher *R*^2^ implies better accuracy.

### Datasets

Six well-known real-world datasets are loaded to evaluate the performances of the FCM-FN model compared with FCM model and FCM-PSO model (See Supplementary File - raw dataset).

Table 5 is a summary of the 6 datasets. These datasets are cases proposed in previous studies in various fields including social, material, medical and software. Based on Pearson's and Spearman’s Correlation Coefficients, only top 3 attributes and the prediction target of each database are selected as simplified benchmark dataset to evaluate the performance. The number of instances is the rows of original databases, and the number of unique instances is the rows of benchmark dataset after deleted the duplicated instances.

**Table 5:** Dataset characteristics.

	White wine	Red wine	Concrete	Boston	Diabetes	QUES
Number of instances	4898	1599	1030	506	442	71
Number of unique instances	3823	1341	1001	506	442	69
Number of attributes	12		9	13	11	11
Top 3 attributes (based on Pearson's and Spearman’s Correlation Coefficients)	Alcohol		Cement	LSTAT	bmi	MPC
	Volatile acidity		Water	RM	s5	RFC
	Citric acid		Age	INDUS	bp	SIZE1

White wine dataset and red wine dataset were obtained from the same source and with the same structure^41^. The two datasets were related to white and red variants of the Portuguese "Vinho Verde" wine. Both datasets contain chemical analysis of wine derived from three different cultivars grown in the same region. Eleven physicochemical attributes of wine were included: 1—fixed acidity, 2—volatile acidity, 3—citric acid, 4—residual sugar, 5—chlorides, 6—free sulfur dioxide, 7—total sulfur dioxide, 8—density, 9—pH, 10—sulphates, 11—alcohol. The prediction target was wine quality, which was graded between 0 (very bad) and 10 (very excellent) by wine experts.

Concrete dataset recorded 1030 instances and 9 attributes. The prediction target was concrete compressive strength, and the 8 input variables including 1—cement, 2—blast furnace slag, 3—fly ash, 4—water, 5—super plasticizer, 6—coarse aggregate, 7—fine aggregate and 8-age. The concrete compressive strength was a highly nonlinear function of age and 7 ingredients. And the actual concrete compressive strength (MPa) for a given mixture under a specific age (days) was determined from laboratory^42^.

Boston dataset recorded 506 house prices. The dataset was taken from the StatLib library which was maintained at Carnegie Mellon University. The original dataset contained 14 attributes. In order to avoid racism, only 13 attributes was contained in the newly version dataset: 1—CRIM (per capita crime rate by town), 2—ZN (proportion of residential land zoned for lots over 25,000 sq.ft.), 3—INDUS (proportion of non-retail business acres per town), 4—CHAS (Charles River dummy variable), 5—NOX(nitric oxides concentration), 6—RM (average number of rooms per dwelling), 7—AGE (proportion of owner-occupied units built prior to 1940), 8—DIS (weighted distances to five Boston employment centres), 9—RAD (index of accessibility to radial highways), 10—TAX (full-value property-tax rate per $10,000), 11—PTRATIO (pupil-teacher ratio by town), 12—LSTAT (lower status of the population), 13—MEDV (Median value of owner-occupied homes in $1000's). And MEDV was usually the prediction target.

Diabetes recorded 442 patients health conditions by 11 attributes: 1—age, 2—sex, 3—bmi (Body mass index), 4—bp (Average Blood Pressure), 5—s1, 6—s2, 7—s3, 8—s4, 9—s5, 10—s6, 11—outcome. And s1–s6 were 6 serum test data one year later. The prediction target was outcome (quantitative indicator of diabetes one year later).

QUES database was selected from popular object-oriented maintainability datasets which were published by Li and Henry^43^. Ten attributes were included as input variables: 1—WMC (Weighted method per class), 2—DIT (Depth of the inheritance tree), 3—NOC (Number of children), 4—RFC (Response for class), 5—LCOM (Lack of cohesion of methods), 6—MPC (Message-passing coupling), 7—DAC (Data abstraction coupling), 8—NOM (Number of methods), 9—SIZE2 (Number of properties), 10—SIZE1 (Lines of code). The prediction target was CHANGE (Number of lines changed in the class), which recorded the number of changed lines in the code during a three-year maintenance period. The values of 11 attributes in QUES was collected from 71 classes in a software system.

### Results

In this section, the results of the proposed model on the real-world datasets are presented. The comparison is implemented among FCM model, FCM-PSO model and FCM-FN model. FCM model is only related to the node *N*_*11*_. After the parameters being tuned, FCM model is transformed into FCM-PSO. And after implementing the FN method and PSO tuning, FCM-PSO model is transformed into FCM-FN.

Table 6 summarized the performance *TI* and *PI* of the three models: FCM, FCM-PSO, FCM-FN. *TI* is related to the structure of models directly. The FCM and FCM-PSO have the same structure and *TI* (= 4). By contrast, the FCM-FN model is with non-identity nodes and connections and lower *TI* (= 1). *PI* is related to both the structure and parameters. Though FCM and FCM-PSO have the same structure, but the latter tunes the parameters and then impact *PI,* which is added from 5.021 to 5.660. Due to lower *TI*, the *PI* of FCM-FN is 1.646 and far lower than the other two models.

**Table 6:** Comparison of the performance (TI and PI).

	FCM	FCM-PSO	FCM-FN
*TI*	4	4	1
*PI*	5.021	5.660	1.646

Table 7 are the results of performance *SMAPE* of the three models utilizing the 6 benchmark datasets. The *AI* of each model is appraised with the process of ten-fold cross-validation. Each dataset is randomly shuffled and then divided into 10 partitions. 9 partitions for training and 1 partition for testing. The value of *AI* is the mean value after running each model 10 times, in format of percentage. In Table 7, the ‘%’ is omitted in order to save space, and the values of best accuracy among the three models are bolded.

**Table 7:** The *SMAPE* of the three models after different iterations’ optimization (%).

Dataset	Model	Iterations										
		10	20	30	40	50	100	200	300	400	1000	2000
White wine	FCM	22.985										
	FCM-PSO	22.936	22.366	22.251	22.066	21.999	21.802	21.794	21.728	21.734	21.745	21.743
	FCM-FN	**22.742**	**22.332**	**22.177**	**21.975**	**21.829**	**21.598**	**21.495**	**21.521**	**21.532**	**21.482**	21.478
Red wine	FCM	23.330										
	FCM-PSO	23.241	22.428	**22.102**	**22.079**	22.064	21.811	21.689	21.693	21.710	21.696	21.704
	FCM-FN	**22.934**	**22.292**	22.132	22.080	**22.038**	**21.795**	**21.679**	**21.680**	**21.697**	**21.692**	**21.700**
Concrete	FCM	37.788										
	FCM-PSO	34.224	32.435	30.829	30.235	29.686	28.514	28.007	27.680	27.656	27.252	27.252
	FCM-FN	**33.947**	**32.338**	**30.599**	**30.035**	**29.535**	**28.375**	**27.966**	**27.630**	**27.617**	**27.218**	**27.214**
Boston	FCM	31.084										
	FCM-PSO	24.119	23.601	21.736	21.647	21.409	21.107	20.988	20.923	20.924	20.931	**21.051**
	FCM-FN	**23.585**	**23.271**	**21.646**	**21.552**	**21.276**	**21.001**	**20.809**	**20.808**	**20.839**	**20.820**	21.054
Diabetes	FCM	47.435										
	FCM-PSO	46.865	45.588	45.759	45.390	45.292	45.161	45.055	44.992	45.237	45.266	**44.755**
	FCM-FN	**46.129**	**45.147**	**45.100**	**44.948**	**44.828**	**44.449**	**44.477**	**44.466**	**44.443**	**44.575**	44.796
QUES	FCM	57.642										
	FCM-PSO	**54.893**	54.522	53.539	53.416	51.768	49.411	47.635	47.213	47.530	47.250	46.212
	FCM-FN	55.225	**54.353**	**53.211**	**53.190**	**51.585**	**49.253**	**47.484**	**46.997**	**47.260**	**47.027**	**46.036**

Significant values are in [bold].

In order to explore the effects of different iterations of PSO, 11 different values from 10 to 2000 are set separately as the MaxNumIteration parameter of PSO. The values of *SMAPE* after 11 different iterations are shown in Table 7. It’s obvious that the accuracy of FN-PSO and FCM-PSO is far better than FCM model, and FN-PSO better than FCM-PSO in most instances.

The means of 11 *SMAPE* values of each model is shown in Table 8, as well as the improvements of FCM-FN compared with FCM and FCM-PSO respectively. The accuracy of the FCM-FN model is improved from 5.012% to 30.784% compared with FCM, and from 0.204% to 1.201% compared with FCM-PSO. The average *SMAPE* of each dataset shows that FCM-FN models marginally outperforms FCM-PSO models. This observation is not surprising since FCM-FN model derived most of its initial semantic inputs from the experts’ knowledge.

**Table 8:** Comparison of mean *SMAPE* on the 6 benchmark datasets (%).

	Mean SMAPE			The improvement of FCM-FN compared with	
	FCM	FCM-PSO	FCM-FN	FCM	FCM-PSO
White wine	22.985	22.015	21.833	5.012	0.827
Red wine	23.330	22.020	21.975	5.808	0.204
Concrete	37.788	29.434	29.316	22.420	0.401
Boston	31.084	21.676	21.515	30.784	0.743
Diabetes	47.435	45.396	44.851	5.447	1.201
QUES	57.642	50.308	50.147	13.003	0.320

**Figure 11:**
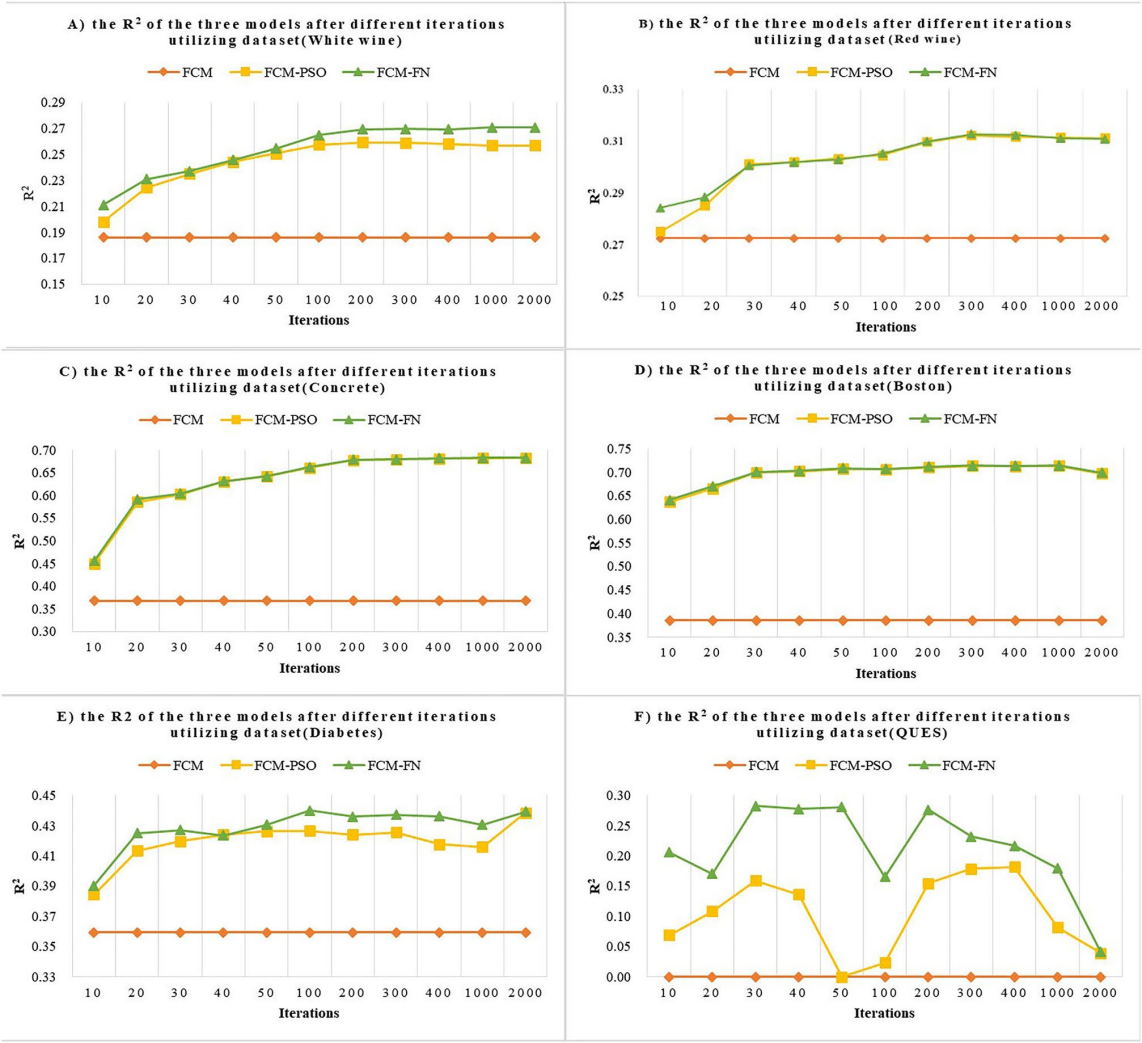
The *R*^2^ of the three models after different iterations’ optimization.

In general, increasing number of iterations in parameter-tuning phase, the *SMAPE* and *R*^2^ is increasing accordingly as shown in Fig. 11A–C and Table 7. But, as shown in Fig. 11D–F, the *R*^*2*^ is not always increasing along with the iterations. What’s more, as shown in Fig. 11F, the *R*^*2*^ fluctuates sharply because there are only 71 instances. As shown in Table 7, the PSO algorithm always converged before reaching the aforementioned MaxNumIteration. For example, The FN-PSO and FCM-PSO reached their best accuracy when the benchmark dataset was red wine and MaxNumIteration was 200, and more MaxNumIteration was not yielded higher accuracy because the FCM-PSO converged no more than 257 iterations and FN-PSO no more than 51 iterations. And doing so increased the duration of the optimization process and also increased validation error due to overtuned system parameters with the training data. Thus, the following accuracy values fluctuated around the best one.

## Conclusion

To improve the performances of prediction model both in accuracy and transparency with well tradeoff interpretability, this paper proposed a FCM-FN model which combines FCM, FN and PSO methods. The main motivation of utilizing FN in prediction model is that interpretability is the dominant feature of a fuzzy model in security-oriented field and other special fields. As noted earlier, FN method has been characterized by the nature of a white-box where the output variable map the input metrics by connections, and the implied information in the FN model has been shown totally. As to the performance of *TI* and *PI*, FCM-FN model is overwhelming compared to FCM model or FCM-PSO model.

Based on FCM method, the numerical data is clustered and then denoted by rules with simple and interpretable structure. Optimized with PSO method, the accuracy of FCM-FN model has been improved significantly (over 5%).

In conclusion, the FCM-FN model has more transparency, interpretability and accuracy than FCM model or FCM-PSO model. Moreover, FCM-FN model is a special type of SFS, because the numerical data and linguistic appraisals can be combined into the prediction model at the same time. Although this strategy is not perfect, it tends to catch more subjective appraisals which have shown their powerful efficacy in previous researches^44,45^.

In this paper only 3 subjective appraisal variables are specified as the example, and the results indicate that it is possible to find qualitative rules. We do not claim that the rules between the 3 variables are the most suitable ones, nor that the FCM-FN based models overwhelm the earlier models in terms of performances other than interpretability and accuracy (*SMAPE* and *R*^2^). Our results showed that FN based models improves the interpretability and accuracy significantly. Notwithstanding its limitation, it is possible to improve the accuracy by implementing more linguistic terms and more accurate subjective appraisals in FCM-FN models. Despite its preliminary character, FCM-FN model is effective for prediction.

To optimize this model in the future, we would like to conduct experiment from the following four aspects: First, integrate type-2 fuzzy system and put it forward in FCM-FN models. Second, integrate other optimization methods, such as GA and “**Patternsearch**”. As an important optimization method in function “**tunefis**”, “**Patternsearch**” performs better for small parameter ranges since they are local optimizers. “**Patternsearch**” may produce faster convergence compared to particleswarm as the tradeoff for accuracy. Third, change the number of linguistic terms or the number of clusters in FCM to explore their influence on accuracy. Forth, integrate newly fuzzy clustering methods to explore their influence on accuracy and interpretability.

## Supplementary Information

Supplementary Information.

## Data Availability

The datasets used and analyzed during the current study are available from three different ways. Three datasets (white wine, red wine and concrete) are available from the UCI Machine Learning Repository (http://archive.ics.uci.edu/ml/index.php), the datasets (boston and diabetes) are available from kaggle website (https://www.kaggle.com/datasets/) and the QUES dataset originally published by Li and Henry^43^.

## Code availability

The MATLAB source code for this project is available upon reasonable request. Please contact Xiaowei Wang (xiaowwei.wang@outlook.com).
